# Alzheimer’s Disease in the Era of Geroscience: Mechanisms, Biomarkers, and Therapeutic Prospects

**DOI:** 10.3390/cells15171599

**Published:** 2026-09-02

**Authors:** Piotr Paweł Chmielewski

**Affiliations:** Division of Anatomy, Department of Human Morphology and Embryology, Faculty of Medicine, Wroclaw Medical University, 6a Chalubinskiego St., 50-368 Wroclaw, Poland; piotr.chmielewski@umw.edu.pl

**Keywords:** Alzheimer’s disease, blood-based biomarkers, cognitive decline, geroscience, gut microbiota, gut–brain axis, insulin resistance, neurodegeneration, neuroinflammation, tauopathy

## Abstract

Alzheimer’s disease (AD) is the leading cause of dementia and a heterogeneous neurodegenerative disorder characterized by amyloid-β (Aβ) and tau pathology, impaired proteostasis, neurovascular dysfunction, maladaptive glial and immune responses, and synaptic dysfunction. Human genetic evidence supports an upstream role for Aβ. Anti-Aβ monoclonal antibodies substantially reduce amyloid burden and modestly slow clinical decline in early symptomatic AD. Continued decline despite plaque removal is consistent with ongoing downstream tau pathology, glial responses, and neuronal injury. This narrative review examines AD mechanisms, biomarkers, and therapeutic prospects from a geroscience perspective and applies the eight hallmarks of neurodegenerative diseases as an analytical framework. Advances in blood-based biomarkers, particularly plasma phosphorylated tau 217, may improve biological detection, but their clinical value depends on assay performance, intended use, and patient context. Gut dysbiosis and gut–brain communication are considered separately as candidate systemic modifiers because causal evidence in humans remains insufficient. The hallmarks are overlapping analytical categories, not independent primary causes, and their therapeutic relevance depends on disease stage and pathway activity. Future studies should establish which preventive strategies and biomarker-guided, stage-matched combination therapies improve clinically meaningful outcomes and identify the patients most likely to benefit.

## 1. Introduction

Alzheimer’s disease (AD) is the most common cause of dementia and a major cause of disability, dependence, and caregiver burden [1,2,3,4]. In 2019, 57.4 million people worldwide were living with dementia, and this number is projected to reach 153 million by 2050, driven largely by population growth and aging [5]. Behind these numbers are progressive losses of memory, cognitive function, and independence, together with substantial burdens on families, health systems, and societies through long-term care, caregiver strain, and lost productivity. Yet the pathological process of AD often begins many years before cognitive impairment becomes clinically evident [6,7,8]. This prolonged preclinical phase creates an opportunity for early detection and intervention, but it also presents a central challenge: the molecular events that initiate amyloid-β (Aβ) and tau pathology may not be identical to the processes that later sustain synaptic dysfunction, neuronal loss, and cognitive decline. Neuropathologically, AD is defined by extracellular Aβ plaques and intracellular tau neurofibrillary tangles and is accompanied by synaptic degeneration and regionally selective neuronal loss [9,10,11,12,13,14,15,16]. Genetic susceptibility, brain reserve, coexisting pathologies, cerebrovascular health, and glial responses influence whether, when, and how this pathology becomes clinically expressed.

A geroscience perspective provides a mechanistic framework for understanding why biological aging increases vulnerability to neurodegeneration and for prioritizing interventions [17]. Normal brain aging is distinct from neurodegenerative disease, and many individuals achieve advanced age free of dementia. Conversely, some older adults remain clinically asymptomatic despite substantial neuropathological burden at autopsy. Nevertheless, advanced age is the strongest risk factor for AD because aging progressively impairs energy homeostasis, mitochondrial function, proteostasis, cytoskeletal integrity, and inflammatory control [11,17]. AD arises on this background of susceptibility, and its lesions further erode homeostatic resilience and lower the threshold for clinical expression. Recently, the field has advanced on several fronts. First, the biomarker framework has been refined, enabling biological diagnosis before dementia onset [6,18]. Second, plasma phosphorylated tau 217 (p-tau217) and related blood-based assays are entering clinical pathways, although performance varies by assay and intended use [19,20,21,22]. Third, lecanemab and donanemab show that substantial reduction in Aβ can modestly slow, but not arrest, cognitive decline in selected patients [23,24]. Taken together, these advances constitute progress while also defining the current limits of diagnosis and treatment.

This narrative review summarizes disease-defining protein pathologies, hallmarks of aging and neurodegeneration, and emerging systemic influences in AD. Risk factors, prevention, and therapeutic approaches are also discussed, along with vascular and metabolic dysfunction, neuroinflammation, and the gut–brain axis.

## 2. From Clinicopathology to Biological Staging

### 2.1. The Amyloid Era and Its Revision

**Figure 1:**
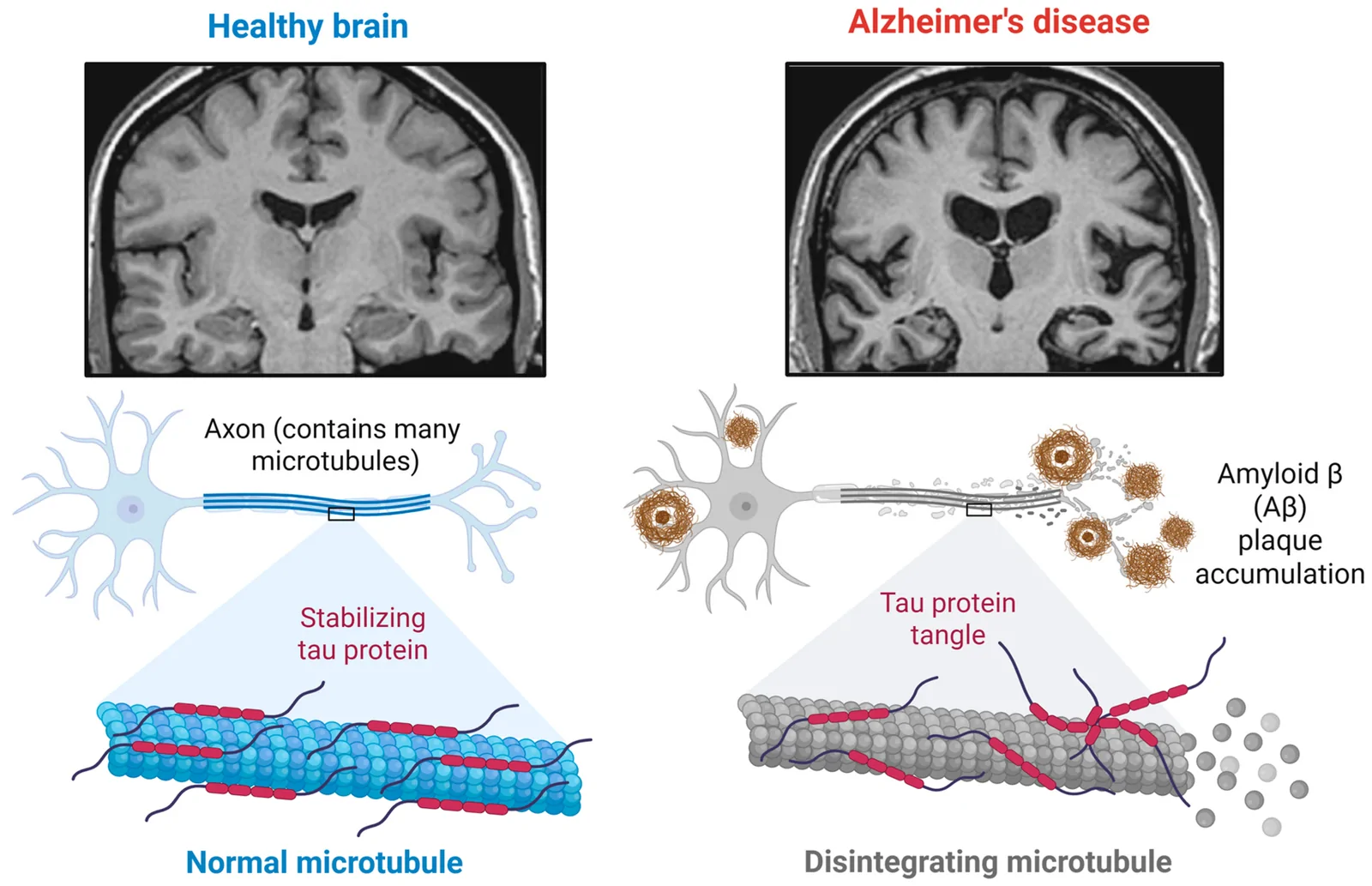
Healthy brain and brain in Alzheimer’s disease (AD). The neuropathological diagnosis of AD is defined by amyloid-β (Aβ) plaques and tau-containing neurofibrillary tangles. These lesions are accompanied by tau-mediated microtubule destabilization, synaptic dysfunction, and neuronal loss, especially in the entorhinal cortex, hippocampus, and amygdala, with later involvement of association cortex, particularly the temporal and parietal cortices.

Alois Alzheimer’s 1906 clinicopathological description linked dementia to two types of microscopic lesions in the brain: extracellular plaques and intracellular neurofibrillary tangles, whose molecular composition was elucidated decades later as aggregates of Aβ peptide and hyperphosphorylated tau filaments, respectively (Figure 1). Pathogenic variants in *APP*, *PSEN1*, and *PSEN2* alter Aβ production or peptide composition, which indicates that aberrant Aβ metabolism can initiate the pathogenesis of AD, especially in autosomal dominant disease, although the mechanistic link between Aβ and tau remains only partially resolved [25,26,27]. The protective *APP* A673T variant reduces β-secretase cleavage of *APP* and provides human genetic evidence that lifelong reduction in Aβ generation can lower the risk of AD and may attenuate age-related cognitive decline [28].

In late-onset AD, *APOE* ε4 strongly modifies risk and age at onset, while genome-wide association studies converge on lipid handling, endosomal trafficking, and innate immune pathways [29,30,31,32,33,34,35]. These data argue against a purely epiphenomenal interpretation of Aβ. Indeed, the amyloid cascade hypothesis remains most persuasive when formulated as a disease-initiation model, rather than a complete linear account of dementia [25,26,27,36].

Most common susceptibility loci identified through genome-wide association studies (GWAS) have individually small effects, and most associated variants are noncoding and exert cell-type-dependent regulatory effects [32,33,34,35]. Genetic associations alone do not establish therapeutically tractable targets. Moreover, polygenic risk estimates derived predominantly from European-ancestry cohorts have limited transferability across ancestries [37].

Aβ accumulation usually precedes widespread neocortical tau pathology and cognitive impairment, but plaque burden may plateau as tau, synaptic dysfunction, and neurodegeneration continue to progress [7,8,38,39,40,41]. Aβ is therefore most defensibly positioned as an upstream pathology that enables or accelerates downstream disease in a manner conditioned by cellular context, anatomical connectivity, and disease duration. The modest clinical effects of plaque-lowering antibodies do not invalidate Aβ causality. These findings are consistent with the view that removing an upstream driver after substantial tau propagation, synaptic loss, and neuronal injury have developed is unlikely to reverse the disease process.

### 2.2. The Cellular Phase and Mixed Pathology

The “cellular phase” model proposes that a long interval separates early protein dyshomeostasis from overt neurodegeneration [9]. During this interval, microglia, astrocytes, neurons, and vascular cells initially adapt to pathology. Their responses may contain plaques, clear debris, maintain synapses, and preserve circuit function. With chronic exposure and aging, however, compensatory programs can become ineffective. The same pathway can therefore be protective at one stage and harmful at another, an important explanation for why nonspecific anti-inflammatory or late-stage interventions have disappointed.

Older human brains frequently contain vascular lesions, α-synuclein pathology, limbic-predominant age-related TDP-43 encephalopathy (LATE), hippocampal sclerosis, and other co-pathologies [1,2,42,43]. These lesions alter clinical expression and reduce the specificity with which a dementia syndrome predicts AD neuropathology. Biological AD should consequently be distinguished conceptually from the clinical syndrome in which it manifests. AD can be present in the absence of symptoms, and dementia may reflect the combined effects of multiple pathologies.

### 2.3. From AT(N) to Revised Biological Criteria

The 2018 NIA–AA Research Framework organized biomarkers into amyloid (A), tau (T), and neurodegeneration or neuronal injury (N), separating disease-defining pathology from clinical stage [6,40]. The 2024 Alzheimer’s Association criteria retain a biological definition but expand biomarker groups and distinguish diagnostic biomarkers from those used to stage severity or characterize non-AD processes [18]. This revision reflects major advances in blood biomarkers and recognizes that A, T, and N are not simple, synchronous, or mechanistically equivalent compartments.

Biological criteria improve research cohorts, trial enrichment, and mechanistic precision, but they require disciplined clinical use [44]. Biomarker results can address defined questions, including whether a specific pathology is likely present, how advanced it may be, or whether a treatment engaged its target. They do not independently establish the cause of a patient’s symptoms, prognosis at the individual level, or appropriateness of treatment. The value of biological diagnosis is therefore greatest when coupled to clinical phenotype, functional trajectory, competing pathologies, and a decision that the result can meaningfully inform [18,21,43,44,45,46].

## 3. Molecular and Cellular Pathogenesis

### 3.1. Aβ Production, Assembly, and Clearance

Sequential cleavage of amyloid precursor protein by β-secretases and γ-secretases generates Aβ peptides of different lengths and aggregation propensities. Aβ42 is particularly prone to assembly, but biologically relevant species include soluble oligomers, protofibrils, insoluble fibrils, and vascular deposits. Soluble assemblies can perturb glutamatergic signaling, calcium homeostasis, membrane integrity, and long-term potentiation before extensive neuronal loss [13,14,25,26,27,47]. Plaques are accompanied by dystrophic neurites, activated glia, complement components, and changes in the extracellular matrix, and their biological effects cannot be inferred from plaque quantity alone.

Net Aβ accumulation reflects production, aggregation, transport, enzymatic degradation, and perivascular fluid clearance. *APOE* genotype, sleep–wake state, vascular pulsatility, blood–brain barrier (BBB) transport, and endosomal–lysosomal function influence this balance [16,29,30,48,49]. Cerebral amyloid angiopathy adds a clinically important vascular dimension and probably contributes to the risk of amyloid-related imaging abnormalities (ARIA) during antibody treatment. Nevertheless, Aβ burden does not determine cognitive status on its own. Some cognitively unimpaired older adults remain stable for years despite amyloid positivity, whereas others progress, particularly when tau pathology, neurodegeneration, or vascular injury is also present [7,41,50,51]. Aβ positivity therefore changes the probability and biological trajectory of disease without determining an individual clinical outcome.

### 3.2. Tau: Regional Vulnerability and Network Propagation

Tau pathology correlates more closely with regional atrophy, symptom severity, and clinical progression than does Aβ plaque burden, likely because tau is more proximate to neuronal injury, synaptic failure, and network degeneration [38,52,53,54,55,56]. In AD, abnormal phosphorylation and other post-translational modifications alter tau conformation, localization, and microtubule binding, thereby contributing to cytoskeletal and axonal-transport dysfunction. This toxicity is probably not confined to mature tangles. Soluble and seed-competent tau species may propagate pathology and contribute to neuronal injury by impairing axonal transport, mislocalizing to dendrites, and disrupting cellular proteostasis. Neuropathological studies indicate that tau pathology follows the stereotyped, though not invariant, anatomical sequence described by Braak and Braak, beginning in the transentorhinal and entorhinal cortices, spreading to the hippocampal formation and the amygdala, and later involving the association neocortex, particularly the temporal, parietal, and frontal association cortices [38,39]. Experimental imaging data support connectivity-constrained propagation, although a “prion-like” spread remains a mechanistic analogy [11,39,53,54,55,56]. Aβ appears to facilitate neocortical tau spread, while local cell identity, network activity, myelination, and metabolic demand influence selective vulnerability. Once tau dissemination and neuronal loss are established, Aβ removal alone is unlikely to reverse the disease.

### 3.3. Microglia, Astrocytes, and Neuroinflammation

Human genetics places innate immunity near the center of late-onset AD biology. Risk variants in *TREM2* and loci related to phagocytosis, lipid metabolism, and complement implicate microglial function [31,32,33,34,35]. Microglia adjacent to plaques adopt transcriptional and metabolic states that differ from homeostatic microglia [57,58]. TREM2–APOE signaling promotes aspects of this transition, affecting lipid sensing, proliferation, plaque compaction, and the response to injured axons [57,58,59,60]. These states are context-dependent. A competent microglial barrier can compact plaques and limit peri-plaque axonal damage [59]. Conversely, persistent inflammasome signaling, reactive oxygen species (ROS) production, cytokine release, and complement-mediated synapse elimination can amplify injury [15,60,61,62,63]. Binary M1/M2 terminology is therefore inadequate, because microglial phenotypes in vivo are multidimensional, dynamic, and regionally heterogeneous. Consequently, therapeutic modulation should aim to preserve beneficial surveillance and repair while attenuating specific injurious programs, rather than indiscriminately suppressing microglia.

Astrocytes similarly regulate synaptic metabolites, glutamate uptake, lipid trafficking, BBB function, and inflammatory signaling. Reactive astrocyte states may be adaptive around acute injury but harmful when they sustain cytokine and complement cascades or fail to support neuronal metabolism [15,62,63]. Oligodendrocyte and myelin dysfunction, although less developed therapeutically, may alter network synchrony and axonal energy supply. Thus, the inflammatory phenotype of AD emerges from interacting glial and vascular compartments rather than a single cytokine pathway.

Microglial aging adds a further layer. Senescence-like, dystrophic, lipid-laden, and disease-associated microglial states share features but are not interchangeable [64,65]. No single marker identifies senescent microglia in the living human brain, and conventional senescence criteria are difficult to apply to long-lived, regionally self-renewing cells with low basal proliferation. Evidence supports age-related loss of phagocytic flexibility, lysosomal dysfunction, altered lipid metabolism, and pro-inflammatory secretory programs, but the contribution of cellular senescence to human AD remains incompletely understood [64]. Consequently, while senolytics and senomorphics hold therapeutic promise, these senotherapeutic approaches are still preliminary and clinically experimental.

### 3.4. Neurovascular, Metabolic, and Environmental Drivers of Neurodegeneration

The neurovascular unit, comprising endothelial cells, pericytes, vascular smooth muscle cells, astrocytic endfeet, microglia, and neurons, couples local blood flow to neural activity and regulates molecular exchange [16,48,66]. BBB breakdown, pericyte injury, arterial stiffness, small-vessel disease, and impaired neurovascular coupling can reduce substrate delivery, impede waste clearance, and expose brain tissue to peripheral inflammatory mediators [16,48,49,66]. Aβ and vascular dysfunction reinforce one another: vascular injury impairs Aβ clearance, while vascular Aβ and inflammatory responses further compromise vessel integrity [16,48,49].

**Figure 2:**
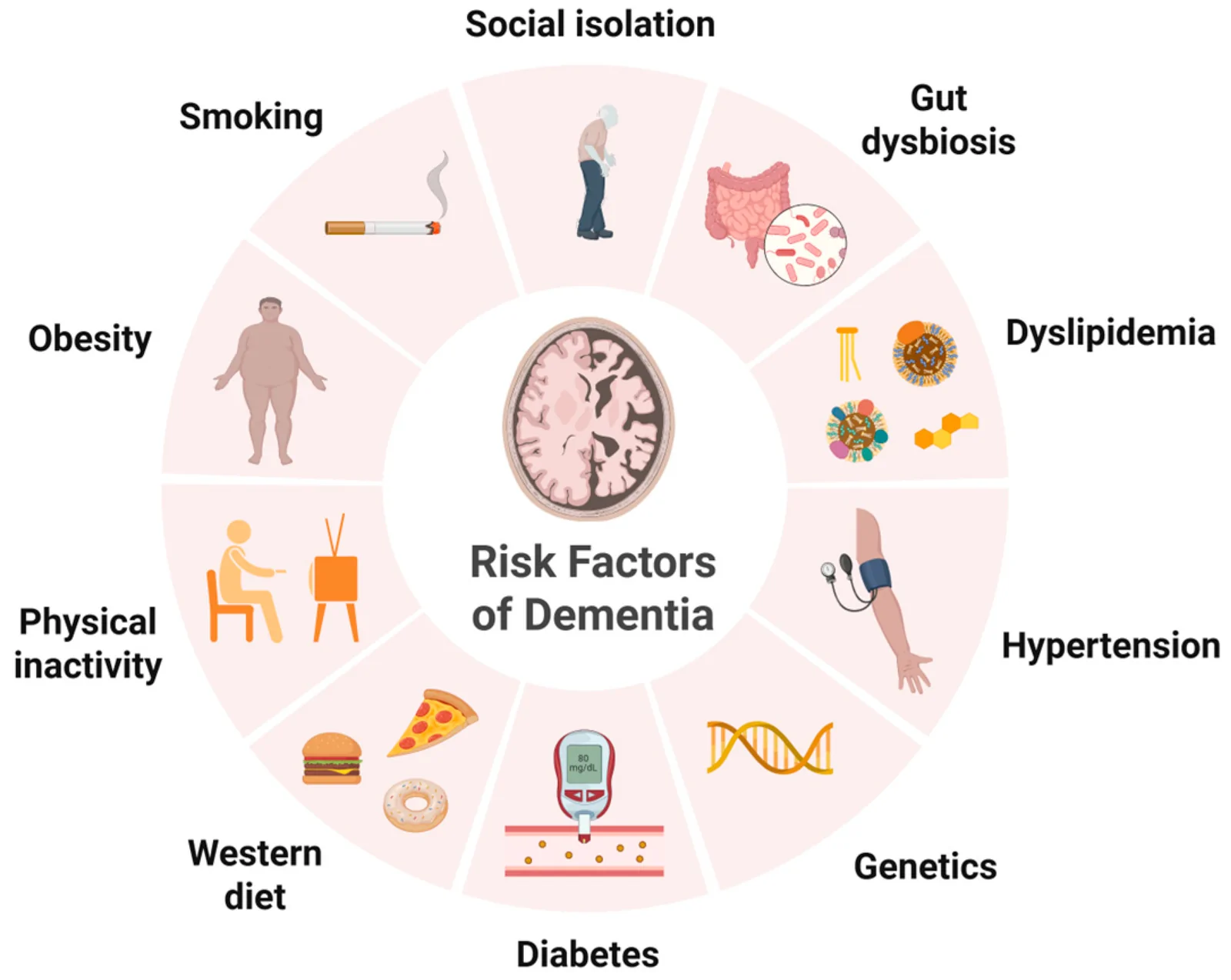
Selected established risk factors and candidate modifiers of dementia. Genetic susceptibility is nonmodifiable. Gut dysbiosis remains a candidate modifier, whereas Western dietary patterns and dyslipidemia act mainly through vascular and metabolic pathways. The diagram does not imply that each factor independently causes Alzheimer’s disease.

Vascular, metabolic, environmental, social, and lifestyle-related risk factors can increase the risk of dementia through partly overlapping pathways that include cerebrovascular injury, systemic inflammation, and reduced cognitive reserve (Figure 2). The 2024 Lancet Commission added elevated low-density lipoprotein cholesterol and untreated vision loss to 12 previously recognized potentially modifiable dementia risk factors [3,67,68,69,70]. While these population-level associations support risk reduction, they do not show that any single exposure initiates AD or alters its defining Aβ and tau pathology. Brain insulin resistance and neuroinflammation may contribute to neurodegeneration, but the term “type 3 diabetes” might oversimplify AD by obscuring its defining protein pathology [68,69,71]. Insulin signaling modulates neuronal and glial metabolism, lipid handling, synaptic plasticity, and inflammatory pathways, but cerebral glucose uptake is largely insulin independent. Systemic metabolic dysfunction has not been established as a primary etiological factor in AD, although it may increase vulnerability to cognitive decline.

### 3.5. Synaptic Dysfunction, Aberrant Proteostasis and Bioenergetics

Synapse loss is among the strongest pathological correlates of cognitive impairment [12,13,14]. It arises from convergent insults: soluble Aβ and tau species, aberrant neuronal activity, complement-dependent pruning, axonal transport defects, mitochondrial dysfunction, and failure of local protein quality control. Network hyperexcitability may appear early and contribute to activity-dependent Aβ release or tau spread, whereas later network failure reflects disconnection and cell loss.

Endosomal trafficking, autophagy, lysosomal degradation, the ubiquitin–proteasome system, and chaperone networks normally prevent the accumulation of damaged proteins and organelles [72,73]. Decline in these systems can become self-reinforcing: aggregates burden clearance mechanisms, impaired clearance increases aggregate load, and damaged mitochondria elevate oxidative stress and reduce ATP supply [72,73,74,75]. Neurons are particularly vulnerable because of their longevity, polarized architecture, high energy demand, and limited regenerative capacity. These mechanisms connect disease-specific lesions to the broader aging-related decline in cellular maintenance.

### 3.6. Sex and Gender as Modifiers

Women account for more AD cases partly because they live longer than men, but observed differences cannot be reduced to longevity alone. Sex can modify *APOE* ε4-associated risk, tau burden, inflammatory and metabolic responses, and biomarker trajectories, although estimates vary by age, hereditary factors, disease stage, and study design [76]. In the WHIMS randomized trial, conjugated equine estrogen plus medroxyprogesterone acetate initiated in women aged 65 years or older increased the incidence of probable dementia [77]. No randomized trial has established menopausal hormone therapy as an intervention to prevent dementia. Education, literacy, and occupational complexity may modify the relationship between neuropathological burden and clinical expression. These variables should be modeled as potential modifiers [76]. Sex differences in response to anti-Aβ antibodies have been suggested by subgroup analyses of several trials.

## 4. Biomarkers and Clinical Staging

### 4.1. What Biomarkers Measure

Biomarkers should be interpreted according to the biological analyte and intended use. A low cerebrospinal fluid (CSF) Aβ42/40 ratio or positive amyloid PET indicates cerebral amyloid pathology [47,78]. CSF phosphorylated tau species reflect AD-related tau pathophysiology, while tau PET provides spatial information about fibrillar tau burden [52,53,54,55,56]. CSF total tau and neurofilament light (NfL) are markers of neuronal or axonal injury but are not specific to AD. Structural magnetic resonance imaging (MRI) and fluorodeoxyglucose PET measure downstream neurodegeneration or dysfunction and can help stage severity and identify alternative causes of cognitive impairment.

A marker can be diagnostically specific without tracking short-term clinical change, prognostic without being disease-specific, or pharmacodynamic without predicting benefit. Composite schemes such as AT(N) are useful organizing languages, not causal models, and different assays within a category do not need to provide interchangeable information [6,18,40,79].

### 4.2. Blood Biomarkers: Opportunity with Guardrails

Plasma p-tau217 is currently the best-validated blood marker of AD; in well-characterized cohorts, high-performing assays approach the diagnostic accuracy of established CSF tests for amyloid and tau PET status [20,22]. Plasma p-tau181 and p-tau231 are informative but show different stage sensitivity and diagnostic performance. GFAP captures an astroglial response associated with amyloid pathology, whereas NfL reflects neuroaxonal injury across numerous neurological diseases [19,20,21,22,80,81,82,83]. Ratios and two-cutoff strategies can define zones of high diagnostic confidence while reserving confirmatory testing for intermediate results.

Clinical implementation should rest on analytical and clinical performance rather than on the analyte name alone. The 2025 Alzheimer’s Association guideline addresses patients with objective cognitive impairment evaluated in specialist care. It recommends that a blood test used to triage patients achieve at least 90% sensitivity and 75% specificity, and that a test substituting for amyloid PET or CSF achieve at least 90% sensitivity and 90% specificity in the intended-use population [21]. These thresholds do not imply that every commercial p-tau test performs adequately. Assay platform, cutoffs, pre-analytical handling, renal dysfunction, age, body composition, and pretest probability can alter interpretation [19,21,81,82].

Blood-based biomarkers should not currently be deployed as standalone population screening for asymptomatic adults. In low-prevalence populations, even good specificity can produce substantial false-positive results, and the psychosocial and therapeutic consequences of identifying preclinical biological AD require careful counseling. In symptomatic patients, results belong within a complete evaluation that addresses history, objective cognition, function, medication effects, mood, sleep, sensory impairment, cerebrovascular disease, and non-AD neurodegeneration [18,21,43,44,45,46].

Major implementation questions remain. Assay-specific cutoffs require external validation, quality-control programs, and calibration in the intended clinical population. Performance in specialist cohorts cannot be assumed in primary care, where multimorbidity and lower pretest probability are common [84]. Before testing, patients should be informed about indeterminate results, incidental biological AD, uncertain individual prognosis, possible anxiety, and potential insurance or employment consequences [84,85]. Biomarker-guided treatment currently extends mainly to amyloid confirmation and safety monitoring for anti-Aβ therapy. Evidence that broader biomarker panels improve treatment selection remains limited. Validation across ancestrally, racially, ethnically, socioeconomically, and geographically diverse populations is essential because existing genetic and biomarker cohorts remain unrepresentative [37,84].

### 4.3. Staging, Prognosis, and Treatment Monitoring

Biomarker trajectories are nonlinear. Amyloid markers change early and may reach a relative plateau, soluble p-tau rises in association with amyloid, tau PET becomes increasingly informative as topographic spread occurs, and injury markers often relate more directly to clinical progression [7,8,86]. Individual trajectories nevertheless vary, and mixed pathology weakens deterministic forecasts. For anti-Aβ treatment, biomarkers serve distinct roles: confirmation of amyloid pathology for eligibility, MRI to assess hemorrhagic risk and monitor ARIA, and amyloid measures to document target removal. A reduction in plaque signal is not itself proof of clinically meaningful benefit. Future trials should link molecular target engagement to downstream effects on tau, inflammation, neurodegeneration, cognition, and function. The goal is not the maximum number of biomarkers, but a parsimonious panel that resolves a decision.

### 4.4. Digital and Multimodal Tools

Digital cognitive measures, wearable sensors, multimodal omics, and machine-learning models may improve longitudinal phenotyping and trial enrichment. However, no digital or artificial-intelligence system currently replaces clinical assessment or validated pathology biomarkers. External validation, calibration across populations and devices, data privacy, algorithmic bias, and demonstration that model outputs improve patient outcomes are prerequisites for clinical use [87].

## 5. Established Prevention, Symptomatic Care, and Approved Disease Modification

### 5.1. Risk Reduction Across the Life Course

The 2024 Lancet Commission identified 14 risk factors for dementia: limited education, hearing loss, hypertension, smoking, obesity, depression, physical inactivity, diabetes, excessive alcohol use, traumatic brain injury, air pollution, social isolation, elevated low-density lipoprotein (LDL) cholesterol, and vision loss [70]. Under the Commission’s counterfactual assumptions, their combined population-attributable fraction was approximately 45% [70]. This estimate does not mean that 45% of individual cases are preventable, that all exposures can be eliminated, or that the effect of changing one factor is independent of the others. Most of these factors are clinically and socially actionable at both the individual and population levels, but population-attributable fractions remain sensitive to residual confounding, exposure clustering, causal assumptions, and heterogeneity in intervention evidence. The FINGER randomized trial showed that a multidomain program combining diet, exercise, cognitive training, and vascular risk monitoring can improve cognitive performance in at-risk older adults [88]. However, the trial did not establish prevention of AD. Coordinated vascular and lifestyle management supports cognitive resilience and is best framed as a foundational risk-reduction strategy that complements disease-specific therapy [3,67,70,88]. Sleep disruption may exacerbate Aβ and tau pathology, although directionality is complex because prodromal neurodegeneration can also impair sleep. Dietary patterns are associated with cardiovascular, metabolic, and cognitive health, yet no specific food or supplement has been established to prevent AD [70].

No validated algorithm currently matches an individual lifestyle prescription to genotype or biomarker profile, and evidence that lifestyle programs interact synergistically with disease-modifying drugs is insufficient. Implementation should therefore emphasize feasible control of established vascular and sensory risks, sustained adherence, and equitable access rather than claims that a personalized lifestyle regimen prevents AD.

### 5.2. Symptomatic Treatment and Comprehensive Care

Cholinesterase inhibitors and memantine provide modest symptomatic benefit for some patients without removing the underlying pathology [1,2]. Treatment of neuropsychiatric symptoms, management of sleep and sensory deficits, caregiver education, advance-care planning, and access to social support remain central to care.

### 5.3. Anti-Aβ Antibodies: Established Disease Modification and Its Limits

Lecanemab and donanemab slow decline in clinical outcomes in biomarker-confirmed early symptomatic AD while reducing amyloid plaque burden [23,24,89,90]. In Clarity AD, the adjusted between-group difference in the 18-month change on the 18-point Clinical Dementia Rating–Sum of Boxes (CDR-SB) was −0.45 points [24]. Donanemab produced statistically significant but similarly modest benefits over 76 weeks. In the combined population of TRAILBLAZER-ALZ 2, the between-group difference was 2.92 points on the 144-point integrated Alzheimer’s Disease Rating Scale and −0.70 points on the CDR-SB, whereas in the prespecified low or medium tau population the corresponding differences were 3.25 and −0.67 points [23]. Treatment effects were therefore larger, in relative terms, at an earlier pathological stage. The threshold for clinically meaningful benefit at the individual level remains debated, and extensive plaque removal did not produce commensurate restoration of cognition or function. These agents neither restore lost function nor stop disease progression.

Clinical use requires an appropriate clinical stage, amyloid confirmation, baseline and surveillance magnetic resonance imaging, and discussion of uncertain individual benefit. ARIA rates differ by agent and by ARIA phenotype, so a single pooled figure conveys little. In the pivotal trials, ARIA with edema or effusion occurred in 12.6% of lecanemab-treated participants and 24.0% of donanemab-treated participants, whereas ARIA with hemosiderin deposition occurred in 17.3% and 31.4%, respectively [23,24]. Most events were asymptomatic, but serious and fatal events occurred. Risk is higher with *APOE* ε4, especially homozygosity, and may be influenced by cerebral amyloid angiopathy and antithrombotic therapy. Long-term safety and durability remain uncertain. Meta-analysis has associated several anti-Aβ agents with accelerated changes in brain volume, but whether these changes reflect harmful neurodegeneration, fluid shifts, plaque removal, or ARIA-related effects is unresolved [91]. Infusion logistics, repeated imaging, cost, and specialist capacity restrict access. Amyloid confirmation by positron emission tomography or cerebrospinal fluid analysis, serial magnetic resonance monitoring, and infusion capacity are unavailable across most of the world, so eligibility and safety pathways validated in specialist centers in high-income countries do not describe the settings in which most dementia occurs [70,84]. Cost-effectiveness estimates depend strongly on health-system infrastructure, eligible populations, treatment duration, and assumptions about long-term benefit.

### 5.4. Learning from Therapeutic Failure

Therapeutic failures provide mechanism-specific lessons rather than a uniform verdict on a target class. The γ-secretase inhibitor semagacestat worsened cognitive and functional outcomes and caused systemic toxicity, illustrating the consequences of inhibiting a protease with essential substrates beyond amyloid precursor protein [92]. The BACE inhibitor verubecestat reduced Aβ production but did not improve clinical outcomes, showing that biochemical target engagement can be insufficient or harmful when the target, agent, or disease stage is unsuitable [93]. Aducanumab illustrates a different failure mode: substantial plaque reduction accompanied clinical evidence that did not converge. Its two phase 3 trials reached opposite conclusions for the primary clinical endpoint, and accelerated approval based on an amyloid surrogate occurred despite the absence of consistent clinical evidence [94,95]. Together, these cases show that engagement of the Aβ pathway is not equivalent to reproducible clinical benefit. For plaque-lowering antibodies specifically, amyloid reduction alone is an insufficient basis for regulatory or clinical decisions. Broad anti-inflammatory approaches have not produced consistent clinical benefit, but these trials did not select participants by a defined injurious immune state [62,63]. Initial extracellular tau antibodies have likewise not produced consistent clinical benefit despite evidence of target engagement [96]. Future trials should establish brain exposure, molecular target engagement, stage suitability, biological subgroup, downstream pathway effects, and a clinically interpretable endpoint before attributing failure to an entire mechanistic hypothesis. The 2026 pipeline includes 158 drugs in 192 trials [97], but pipeline breadth is not evidence of efficacy, and adequately controlled combination trials remain uncommon.

## 6. Experimental and Investigational Therapeutic Approaches

### 6.1. Tau, APOE, and Glial-State Interventions

Tau-directed antibodies, aggregation inhibitors, vaccines, and RNA-targeting strategies seek to reduce tau production, seeding, aggregation, or intercellular propagation [52,53,54,55,56,96]. The central translational challenge is selecting the relevant tau species and treating before neuronal loss dominates. Tau PET and fluid tau measures may allow stage enrichment and pharmacodynamic assessment, but no tau-directed therapy has established phase 3 clinical efficacy.

APOE is attractive because it connects amyloid deposition, clearance, lipid transport, cerebral amyloid angiopathy, tau-mediated injury, and glial states [29,30,34,35,60]. Potential approaches include altering APOE abundance, lipidation, receptor interactions, or isoform-specific toxic functions. However, APOE performs essential functions in central and peripheral lipid transport, membrane repair, and cellular responses to injury. Reducing an isoform-specific pathogenic effect without disrupting these functions is therefore a major challenge, and effects may differ by disease stage. Decades of mechanistic work have not yet yielded an approved APOE-directed therapy, and human target engagement and clinical benefit remain unproven. Similarly, TREM2 agonism or complement and inflammasome modulation could reshape microglial responses, but preclinical target engagement should not be conflated with human benefit [15,57,58,59,60,61,62,63].

### 6.2. Geroscience-Based Strategies and Translational Constraints

**Figure 3:**
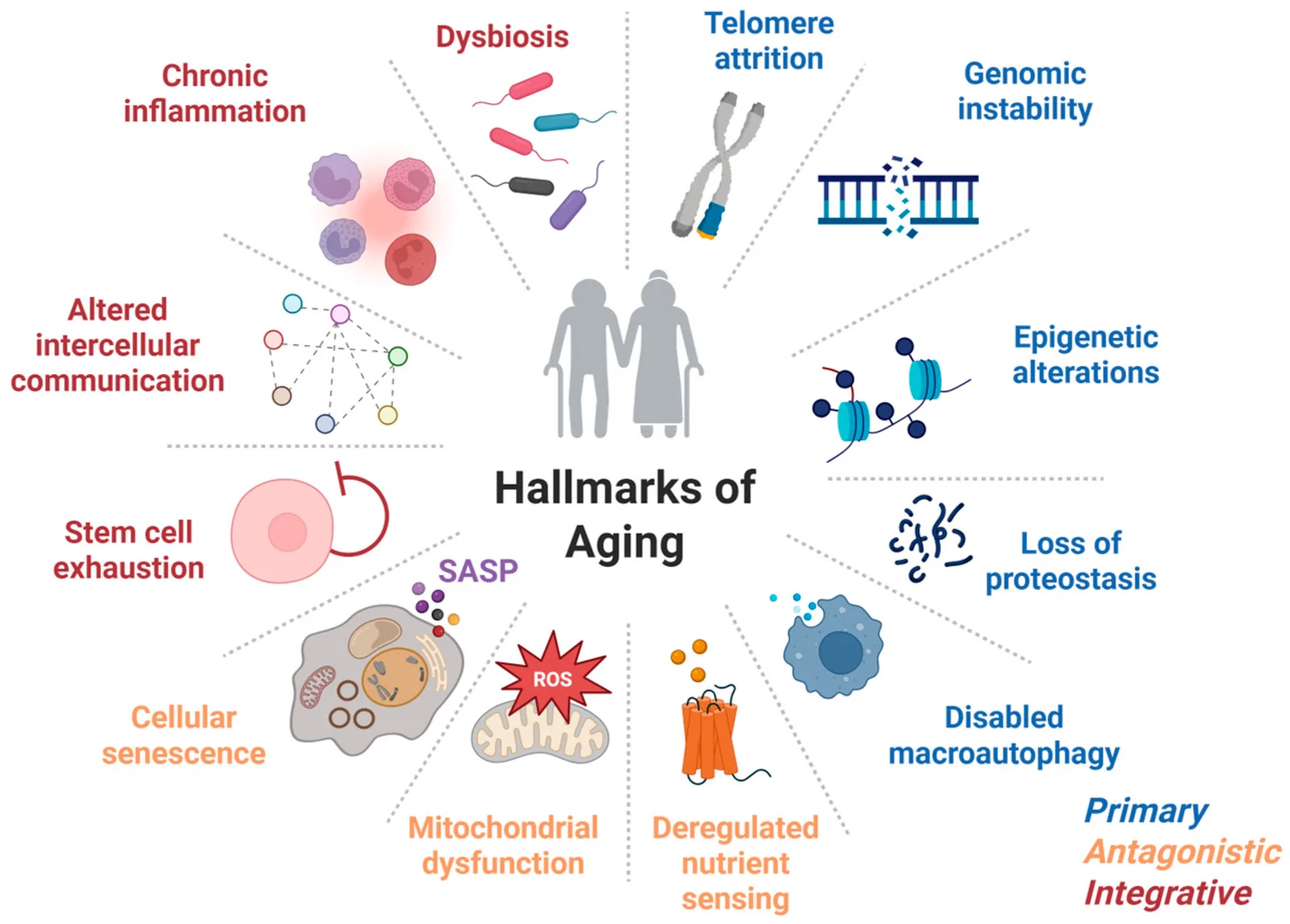
The twelve hallmarks of aging [17]. These interconnected processes contribute to age-related decline in homeostatic capacity and intersect with mechanisms of neurodegeneration. Although they can increase susceptibility to AD, they are neither AD-specific nor equivalent to its defining Aβ and tau pathology.

**Figure 4:**
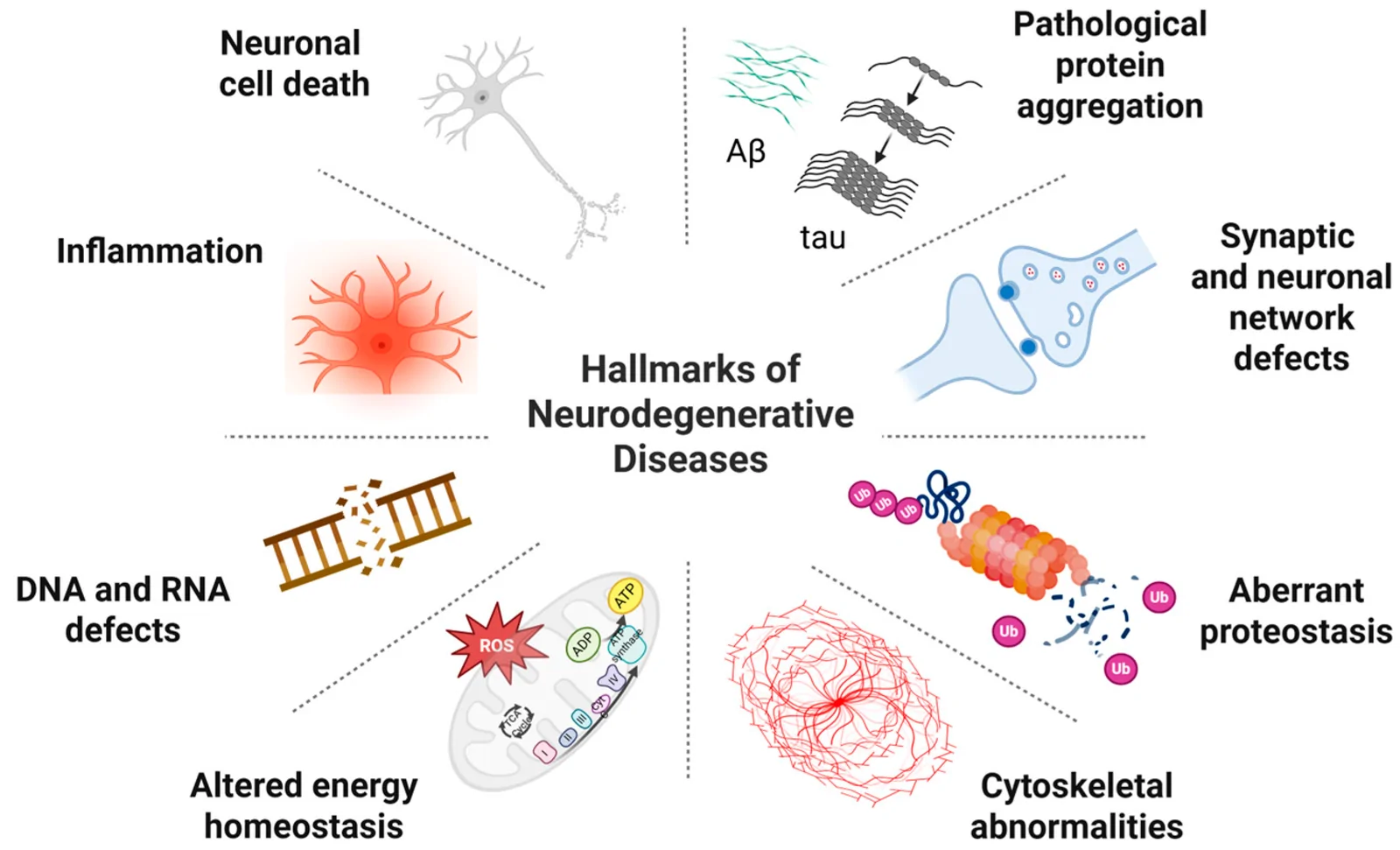
The eight hallmarks of neurodegenerative diseases as an analytical framework for Alzheimer’s disease (AD) [11]. The framework encompasses pathological protein aggregation, synaptic and neuronal network dysfunction, aberrant proteostasis, cytoskeletal abnormalities, altered energy homeostasis, DNA and RNA defects, inflammation, and neuronal cell death. These interacting categories are neither independent nor equally causal. Aβ aggregation has the strongest evidence for an upstream role, whereas tau pathology, synaptic failure, glial responses, metabolic dysfunction, impaired proteostasis, and vascular injury may mediate or amplify progression. Neuronal death is a convergent, irreversible endpoint. The framework can organize mechanisms, biomarkers, and therapeutic hypotheses, but has not been validated for patient stratification or treatment selection.

Geroscience-based approaches ask how aging-associated changes increase susceptibility to neurodegeneration and modify disease progression. The updated hallmarks of aging [17], which include impaired autophagy, loss of proteostasis, mitochondrial dysfunction, and chronic inflammation (Figure 3), intersect with mechanisms of neurodegeneration (Figure 4) [11]. Interventions targeting these processes generate testable hypotheses for disease modification. Some candidate geroscience interventions affect several biological processes simultaneously.

However, broad biological activity also increases the risk of off-target and stage-dependent adverse effects. For example, a small phase 1 study of dasatinib plus quercetin demonstrated feasibility and detected dasatinib in CSF, but it was underpowered and not designed to evaluate clinical efficacy in mild AD [98]. Senotherapeutic approaches therefore remain experimental and require adequately controlled trials. Interventions that preserve BBB integrity, improve neurovascular coupling, or reduce systemic vascular burden could complement protein-directed therapies [16,48,49,66,67,68,69,70,71,99]. Trials should include participants with vascular comorbidities common in clinical practice and use endpoints that distinguish reduced vascular injury from modification of defining AD pathology.

No intervention directed at a generic hallmark of aging has demonstrated disease-modifying efficacy in human AD. Trials in older adults must account for frailty, multimorbidity, altered pharmacokinetics, immune suppression, impaired wound healing, and interactions with standard therapies. Geroscience therefore supplies hypotheses and candidate targets, not a validated substitute for AD-specific causal evidence.

### 6.3. Genetic and RNA-Based Approaches

Dominantly inherited AD offers a strong rationale for mutation-directed prevention, and antisense or gene-regulatory approaches could eventually modulate *APP*, *MAPT*, or *APOE* expression. For sporadic AD, polygenicity, cell-type specificity, brain delivery, durability, immune responses, and irreversible off-target effects make broad gene editing substantially more challenging. Germline editing is not currently an acceptable therapeutic strategy for AD. Near-term translation is more likely to come from reversible RNA modulation or vector-based expression tested in genetically or biomarker-defined subgroups than from permanent editing of common late-onset risk variants.

### 6.4. Combination Therapy

Combination therapy is rational only when each component has an evidence-supported mechanism, a compatible safety profile, and a biomarker-defined therapeutic window. One testable sequence would pair early Aβ lowering with subsequent or concurrent interventions directed at tau propagation, defined neurodestructive glial programs, or vascular injury. However, combining individually plausible agents can produce mechanistic antagonism or cumulative toxicity. Adaptive platform trials, factorial designs, and validated intermediate biomarkers may make combination studies more tractable [97,100,101]. The governing principle should be stage-matched mechanism rather than maximal target count.

## 7. Hallmarks of Neurodegenerative Diseases

### 7.1. The Eight Hallmarks of Neurodegenerative Diseases as a Framework for Alzheimer’s Disease

The cross-disease taxonomy proposed by Wilson and colleagues [11] is applied here as an analytical classification rather than as a second causal account. Three questions are asked of each hallmark: which AD manifestations map to the category, what can be measured in living patients, and what translational inferences are justified. The eight categories comprise pathological protein aggregation, synaptic and neuronal network dysfunction, aberrant proteostasis, cytoskeletal abnormalities, altered energy homeostasis, DNA and RNA defects, inflammation, and neuronal cell death (Figure 4).

The hallmarks are overlapping analytical categories, not independent or equivalent causes of AD. Aβ has the strongest evidence for an upstream role, especially in autosomal dominant disease. Tau, synaptic dysfunction, inflammation, altered energy homeostasis, and impaired proteostasis can mediate or amplify progression, whereas neuronal death is a convergent and irreversible endpoint. The framework may organize biomarkers and therapeutic hypotheses, but its predictive validity, utility for biological subtyping, and capacity to guide individualized treatment remain unestablished. The principal AD manifestations, candidate measures, and translational interpretation of each hallmark are summarized in Table 1.

### 7.2. Pathological Protein Aggregation

Within the hallmark framework, pathological protein aggregation is not a single mechanism. In AD, it includes parenchymal Aβ plaques, vascular Aβ deposits in cerebral amyloid angiopathy, and intracellular tau inclusions. These lesions differ in cellular compartment, anatomical distribution, temporal course, and relationship to clinical impairment. Amyloid PET and CSF or plasma Aβ42/40 establish cerebral amyloidosis, whereas tau PET maps fibrillar tau, whose cortical distribution is more closely related to neurodegeneration and clinical phenotype [19,22,47,52,53,54,55,56,78,79,80,81,82,83]. None of these measures directly quantifies soluble Aβ oligomers or seed-competent tau species, which remain biologically important but incompletely measurable in living patients [13,14,26,27,52,53,54,55,56,96,104,105].

Nevertheless, the therapeutic evidence also requires a lesion-specific interpretation. Lecanemab and donanemab remove substantial amounts of amyloid plaque and modestly slow decline in early symptomatic AD. These findings show that amyloid deposition is modifiable and clinically relevant, but also that plaque burden alone does not account for established clinical progression [23,24,89,90]. Tau topography and burden provide a closer index of downstream disease severity, although tau PET measures fibrillar deposits rather than the complete spectrum of potentially toxic tau assemblies [52,53,54,55,56]. Pathological aggregation should therefore be understood as a set of molecularly and anatomically distinct processes, not as a single burden that can be summarized by one biomarker or targeted uniformly.

### 7.3. Synaptic and Neuronal Network Dysfunction

Synaptic failure is measurable in part because it precedes extensive neuronal death [12,13,14]. Aβ assemblies can impair long-term potentiation, glutamate-receptor trafficking, calcium regulation, and neurotransmitter release. Pathological tau can redistribute from axons to somatodendritic compartments, disturb synaptic signaling, and increase neuronal susceptibility to excitotoxic and metabolic stress [9,10,11,12,13,14]. These mechanisms affect synaptic function before they necessarily produce an observable loss of neurons.

Network dysfunction is spatially and temporally heterogeneous. Hyperexcitability can occur in vulnerable hippocampal and cortical networks during early disease, while progressive synaptic loss produces disconnection and declining network activity at later stages [10,11]. Neuronal activity can influence the extracellular release of Aβ and tau in experimental systems, which creates the potential for feedback between circuit activity and protein pathology. This mechanism is biologically plausible but should not be treated as the sole explanation for anatomical progression in humans.

Glial cells modify synaptic integrity. Microglia remove weak or damaged synapses as part of normal circuit maintenance, but complement-associated pruning can become excessive in the presence of persistent Aβ, tau, and inflammatory signaling. Astrocytic dysfunction can impair glutamate uptake, ion homeostasis, metabolic support, and synaptic remodeling [15,57,58,59,60,61,62,63]. These processes link synaptic dysfunction to inflammation and altered energy homeostasis.

Synaptic dysfunction retains some potential for reversibility before structural disconnection becomes extensive. The modest symptomatic effects of cholinesterase inhibitors and memantine demonstrate that modifying neurotransmission can improve function without altering Aβ or tau pathology [1,2]. Research measures such as synaptic vesicle glycoprotein 2A PET, electrophysiology, functional imaging, and CSF synaptic proteins may improve assessment of synaptic integrity, but none currently provides an AD-specific measure suitable for routine treatment selection [11].

Degeneration is not confined to cortical pyramidal neurons. Cholinergic basal-forebrain neurons, noradrenergic locus coeruleus neurons, and serotonergic raphe neurons are affected across the disease course and can contribute to impaired attention, arousal, plasticity, mood, sleep, and network regulation [106]. Their involvement is heterogeneous and is not specific to AD. MRI and fluid injury markers do not directly count neurons or identify the affected transmitter-defined population. Biomarkers of cell-type-specific neuronal loss therefore remain a research need.

### 7.4. Aberrant Proteostasis

Proteostasis consists of protein synthesis, folding, trafficking, quality control, and degradation. It must be distinguished from protein aggregation because it describes the systems that prevent or resolve proteotoxic stress rather than the aggregates themselves. Neurons depend on molecular chaperones, the ubiquitin–proteasome system, endosomal trafficking, autophagy, and lysosomal degradation to maintain proteins and organelles across long axons and throughout life [11,72,73].

Endosomal abnormalities are among the earliest cellular changes observed in AD. They are mechanistically relevant because amyloid precursor protein processing occurs within endocytic pathways. AD genetic studies also implicate endosomal trafficking, lipid handling, and microglial phagocytic pathways, which connect proteostasis to Aβ metabolism and innate immunity [9,10,33,34,35]. Autophagic vacuoles accumulate within dystrophic neurites, and impaired lysosomal clearance favors the retention of damaged proteins, mitochondria, and other cellular cargo [72,73]. Aβ and tau can further disrupt proteasomal, autophagic, and lysosomal function, creating a feed-forward relationship between aggregation and defective clearance.

Therapeutic manipulation requires greater precision than simply increasing autophagy. Increasing autophagosome formation without restoring lysosomal degradation might intensify cargo accumulation. Effective intervention may instead require correction of the specific defect in cargo recognition, vesicular transport, lysosomal acidification, substrate degradation, or organelle turnover [72,73]. No validated fluid or imaging biomarker currently measures neuronal proteostatic flux in living patients. This limitation complicates target-engagement studies and remains a major obstacle to clinical development [97,100].

### 7.5. Cytoskeletal Abnormalities

The neuronal cytoskeleton includes microtubules, actin filaments, and neurofilaments. These structures maintain axonal and dendritic architecture, support synaptic remodeling, and transport vesicles, RNA, proteins, and mitochondria between the cell body and distant synapses [11]. Cytoskeletal failure in AD therefore extends beyond the presence of neurofibrillary tangles. Tau normally associates with axonal microtubules. Pathological modification, conformational change, aggregation, and redistribution of tau can impair microtubule organization and axonal transport. Loss of normal tau function may coexist with toxic effects of mislocalized and aggregated tau [9,10,11]. Aβ plaques are surrounded by swollen and dystrophic neurites in which cytoskeletal organization, organelle transport, and lysosomal clearance are disrupted. Plaque-associated microglia can limit this local neuritic injury under some conditions, which illustrates the close relationship between aggregation, cytoskeletal damage, and inflammation [59].

Actin remodeling within dendritic spines affects synaptic structure and plasticity, while neurofilament disruption accompanies axonal degeneration. Neurofilament light concentrations in CSF and blood provide a sensitive measure of axonal injury but are not specific to AD and do not identify the responsible cytoskeletal mechanism [19,81,82,83]. Tau PET maps fibrillar tau pathology but does not measure microtubule stability, axonal transport, or normal tau function [52,53,54,55,56]. These distinctions are important because reducing tau aggregation and restoring cytoskeletal function are related but biologically different therapeutic objectives.

### 7.6. Altered Energy Homeostasis

Neurons require continuous energy production to maintain membrane potentials, calcium gradients, axonal transport, neurotransmission, and synaptic plasticity. Mitochondrial dysfunction can reduce ATP production, impair calcium buffering, increase reactive oxygen species, and compromise the removal of damaged mitochondria through mitophagy [72,74,75]. These abnormalities can weaken synapses before neurons die, but their temporal position in sporadic AD remains uncertain. They may precede, accompany, or result from Aβ accumulation, tau pathology, inflammation, and neuronal injury.

Reduced cerebral glucose metabolism is a reproducible feature of symptomatic AD. However, fluorodeoxyglucose PET measures regional glucose uptake rather than mitochondrial ATP production. Its signal is affected by synaptic activity, neuronal integrity, astrocytic metabolism, and tissue composition [1,2,11]. Likewise, energy homeostasis depends on vascular delivery of oxygen and metabolic substrates. BBB disruption, impaired neurovascular coupling, small-vessel disease, and reduced perivascular clearance can interact with Aβ and tau pathology while independently worsening cognitive function [16,48,49,66,67,99]. Insulin resistance, diabetes, and obesity contribute to vascular, inflammatory, and metabolic stress [68,69,70,71]. These conditions can increase the risk of cognitive decline and may also modify the clinical progression of AD. Therefore, control of hypertension, diabetes, physical inactivity, and other metabolic risks is justified and might reduce cognitive decline at the population level [67,68,69,70,88].

### 7.7. DNA and RNA Defects

Inherited defects in DNA repair or RNA metabolism can directly cause several neurodegenerative diseases, which establishes that these processes are capable of driving neuronal degeneration [11]. Their causal status in AD is less certain. Increased oxidative DNA damage, DNA strand breaks, altered repair responses, epigenetic changes, and abnormalities in RNA splicing, transport, stability, and translation have been reported in AD tissue and experimental models [11,17].

However, several limitations are worth noting. First, oxidative DNA damage can arise downstream of mitochondrial dysfunction and inflammation. Second, transcriptomic and splicing abnormalities may reflect altered cellular states, selective neuronal loss, or changes in cell-type composition rather than a primary defect in RNA metabolism. Furthermore, postmortem findings are affected by disease stage, agonal factors, tissue quality, and coexisting pathology. These issues make it difficult to determine whether a given abnormality initiated degeneration, amplified it, or resulted from it.

Thus, the evidence supports DNA and RNA dysfunction as a biologically credible component of AD, but not as an established upstream mechanism in typical sporadic disease. No DNA- or RNA-based measure has been validated for routine AD diagnosis, biological staging, prognosis, or treatment selection. This hallmark should remain in the framework, but its causal rank and clinical utility should be described as unresolved [11,17].

### 7.8. Inflammation

Inflammation in AD comprises dynamic responses of microglia, astrocytes, vascular cells, and peripheral immune pathways. It is neither uniformly protective nor uniformly injurious. Human genetics provides strong evidence that innate immune biology influences AD risk. Rare *TREM2* variants increase risk, while common susceptibility loci implicate microglial function, lipid metabolism, endocytosis, and phagocytosis [31,32,33,34,35]. APOE also connects lipid transport, amyloid deposition, cerebral amyloid angiopathy, tau-mediated injury, and glial responses [29,30,31,32,33,34,35,58,60].

Plaque-associated microglia can compact Aβ deposits and form a barrier that limits the exposure of nearby neurites to diffuse amyloid. Reduced TREM2 function can weaken this response and increase plaque-associated axonal injury [57,58,59,60]. Persistent immune activation can nevertheless become harmful through complement-mediated synapse elimination, inflammasome activation, oxidative stress, altered lipid handling, and inflammatory signaling between microglia and astrocytes [15,57,58,59,60,61,62,63]. The same cell population can therefore perform protective and injurious functions according to its state, location, substrate, and disease stage.

Disease-associated microglial states identified through single-cell studies have improved mechanistic understanding, but states defined in mouse models do not map directly onto human microglial populations [57,58,59,60]. Senescent or senescence-like microglial phenotypes are also under investigation, but their prevalence and causal importance in human AD remain unresolved [64].

Plasma GFAP can detect an astrocytic response associated with AD, while soluble TREM2 and inflammatory imaging ligands provide research measures of microglial activity [19,62,81,82]. These biomarkers do not identify whether the measured response is beneficial or harmful, and none currently selects a proven immune-directed treatment. The failure of nonspecific anti-inflammatory approaches does not establish that inflammation is irrelevant. It indicates that future interventions must target a defined pathway, cell state, and biological stage while preserving essential immune functions [62,63,97,100,107].

### 7.9. Neuronal Cell Death

Neuronal cell death is not an initiating cause of AD. It is the irreversible endpoint of interacting pathological processes and directly produces a permanent loss of circuit capacity. Neuronal dysfunction and neuronal death must be distinguished because dysfunctional neurons and synapses may remain recoverable, whereas dead neurons cannot be restored by removing Aβ or suppressing inflammation [10,11]. Neuronal loss in AD is regionally selective. It affects the entorhinal cortex, hippocampal formation, and association cortices more severely and earlier than many primary sensory and motor regions. A clinicopathological study of 17 histologically confirmed cases found that dementia severity correlated more strongly with abnormalities of large cortical neurons, including cell loss and reductions in nuclear, nucleolar, and cytoplasmic RNA measures, than with cortical plaque or tangle frequency [102]. Separately, a quantitative stereological study of the superior temporal sulcus, a high-order association cortex, found more than 50% neuronal loss in AD and showed that neuronal loss exceeded the number of tangle-bearing neurons by manyfold [103]. This result indicates that neuronal loss is not restricted to tangle-bearing neurons. Tau distribution is more closely related than amyloid plaque burden to the topography of atrophy and the clinical phenotype [1,2,52,53,54,55,56]. This relationship does not prove that fibrillar tau is the only cause of neuronal death. It shows that tau pathology is closely associated with the anatomical progression of degeneration.

Apoptotic and necrotic mechanisms, as well as necroptosis, ferroptosis, pyroptosis, and other regulated death programs, have been implicated in experimental models and human tissue [11]. Yet no single program has been established as the dominant mechanism of neuronal death in human AD. MRI measures tissue atrophy rather than neuronal death directly. Atrophy can reflect neuronal, dendritic, synaptic, axonal, and glial changes. Neurofilament light measures axonal injury, while total tau is also a nonspecific injury marker. Neither identifies a particular death pathway [18,19,81,82,83]. The irreversibility of neuronal loss explains why therapeutic timing matters. In early symptomatic AD, anti-Aβ antibodies can slow further clinical decline but cannot reconstruct circuits that have already been destroyed [23,24,100]. At later stages, care therefore remains predominantly symptomatic and supportive. Any future restorative intervention would need to replace or compensate for lost circuit function in addition to controlling upstream pathology.

### 7.10. Hallmark Interactions, Selective Vulnerability, and Clinical Implications

The categories are also linked by feedback, so downstream responses can themselves become drivers of further progression. Protein aggregation can overwhelm endosomal, autophagic, and lysosomal clearance, allowing damaged mitochondria and aggregation-prone proteins to accumulate. Energetic failure impairs axonal transport, calcium homeostasis, and synaptic transmission, whereas cytoskeletal disruption limits organelle delivery to distal compartments. Synaptic injury activates glia, and sustained inflammation can further damage synapses, vessels, and proteostasis [9,10,11,15,48,57,58,59,60,61,62,63,64,65,66,67,68,69,70,71,72,73,74,75]. Selective vulnerability reflects intrinsic differences among neuronal populations and is intensified by aging, cerebrovascular disease, and other proteinopathies [1,2,9,10,11,17].

Translationally, core AD biomarkers should be distinguished from markers of downstream injury or response, as in the 2024 criteria [18]. Biomarker panels are useful only if they improve prognosis, predict treatment response, or identify actionable mechanisms. Therapeutic combinations should therefore follow causal and temporal evidence: amyloid lowering is most plausible before extensive tau accumulation and neuronal loss, whereas tau-, immune-, synaptic-, vascular-, or proteostasis-directed therapies may add benefit only when the relevant pathway is active and target engagement is achieved [23,24,97,100].

## 8. Gut Dysbiosis and Gut–Brain Axis

Microbiome findings are less causally established than the mechanisms considered above, and they are therefore treated separately as a candidate systemic modifier rather than as disease-defining AD pathogenesis.

### 8.1. Aging and Dysbiosis

The updated hallmarks of aging include genomic instability, telomere attrition, epigenetic alterations, loss of proteostasis, disabled macroautophagy, deregulated nutrient sensing, mitochondrial dysfunction, cellular senescence, stem-cell exhaustion, altered intercellular communication, chronic inflammation, and gut dysbiosis [17]. These interconnected processes contribute to biological aging and may create vulnerabilities to neurodegeneration through impaired protein clearance, metabolic dysfunction, and sustained inflammatory signaling.

**Figure 5:**
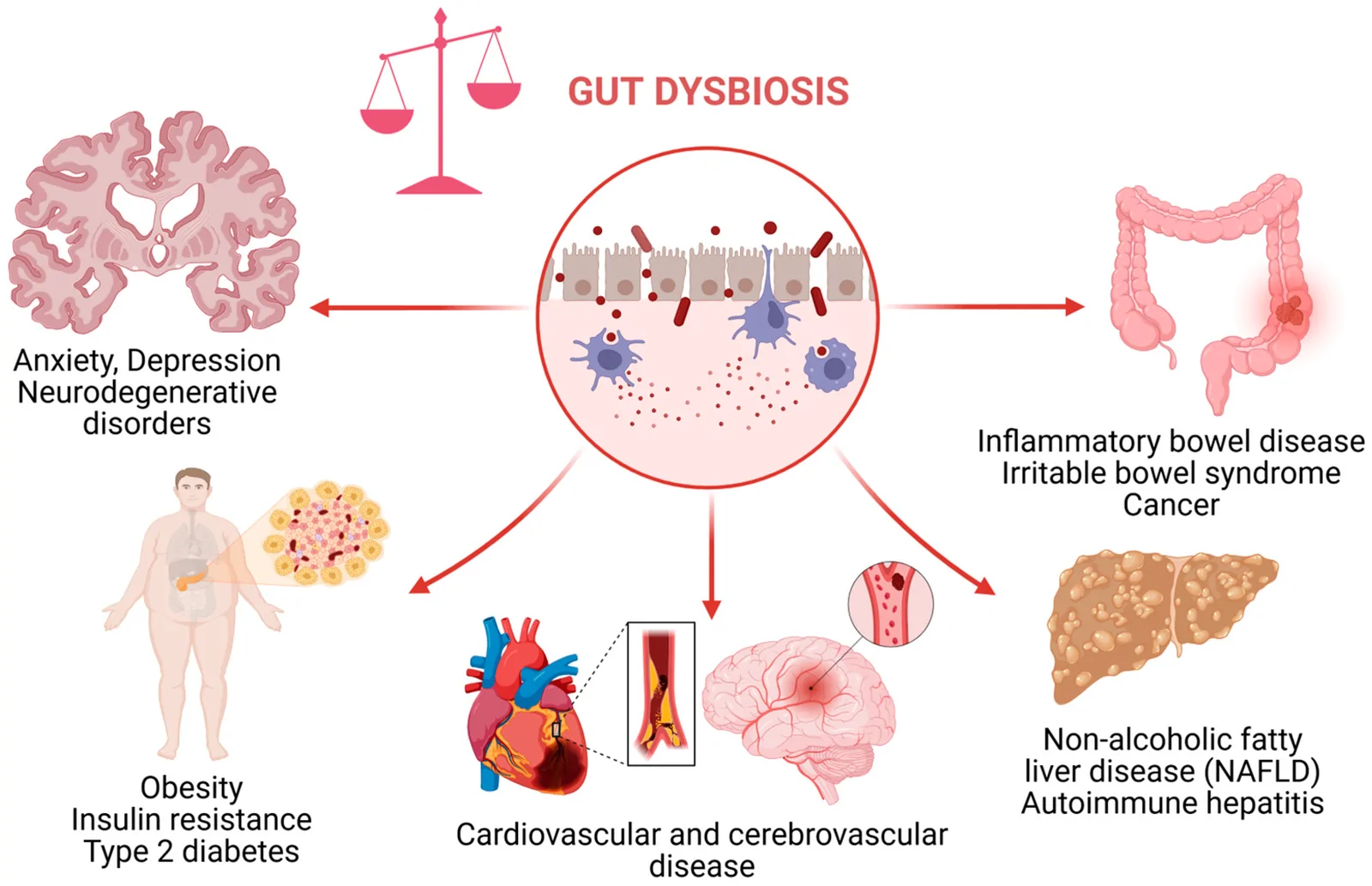
Gut dysbiosis and chronic disease. Dysbiosis has been associated with neurodegenerative, metabolic, cerebrovascular, cardiovascular, intestinal, and liver disorders through partly shared immune, metabolic, barrier, and neural pathways. The arrows indicate reported associations and proposed pathways. They do not establish causality or the direction of the relationship. Microbiome composition and function can also be altered by age, diet, medication, comorbidity, and disease-related changes in behavior and physiology.

Dysbiosis, which is a context-dependent disruption of microbial community structure and function relative to host physiology, has attracted particular attention because of bidirectional gut–brain communication. Gut dysbiosis has been associated with systemic inflammation, altered metabolite production, and compromised BBB integrity, potentially exacerbating neuroinflammation and protein-aggregation pathways relevant to AD (Figure 5). The gut–brain axis includes endocrine, immune, metabolic, and vagal pathways through which microbial communities can affect neuronal function and survival. Nevertheless, causal links between gut dysbiosis and AD pathogenesis in humans remain to be established. Although observational studies associate microbiome alterations with cognitive decline, it remains unclear whether they drive neurodegeneration, arise as a consequence of disease, or reflect shared risk factors. Resolving this question will require longitudinal studies, validated biomarkers, and carefully controlled clinical trials.

### 8.2. The Gut–Brain Axis

Gut–brain communication is bidirectional and occurs through several partially overlapping routes [108]. Microbial metabolites enter host pathways. Intestinal epithelial and immune cells signal through cytokines and endocrine mediators. Vagal and enteric neural circuits convey physiological state, and central autonomic output alters motility, permeability, secretion, and microbial habitat. AD-related changes in behavior and autonomic function can thus reshape the gut while gut-derived signals influence the brain.

Reduced epithelial integrity may increase exposure to microbial products such as lipopolysaccharide, peptidoglycan fragments, or microbial nucleic acids. These signals can activate peripheral innate immunity and endothelium without needing intact microbes to enter the brain. Systemic inflammation may impair BBB function, alter monocyte and neutrophil behavior, and prime microglia [15,48,109,110]. However, circulating endotoxin assays and surrogate permeability markers are methodologically variable, and direct evidence that this route initiates sporadic human AD is lacking.

Short-chain fatty acids (SCFAs) regulate epithelial integrity, immune cell differentiation, and microglial maturation, but their effects depend on concentration, receptor, tissue, and disease context. They should therefore not be described as uniformly neuroprotective. Secondary bile acids, produced through host–microbial co-metabolism, have been associated with cognitive and biomarker phenotypes, while tryptophan metabolism links microbial ecology to serotonin, indole, and kynurenine pathways [110,111]. These associations are biologically plausible but do not yet establish that modifying a single metabolite will alter AD progression.

Vagal afferents and the enteric nervous system sense nutrients, microbial metabolites, and immune mediators [108]. Stress hormones and hypothalamic–pituitary–adrenal activity can reciprocally modify gut permeability and microbial ecology. Evidence for these routes is strong in general physiology and animal models, but the extent to which they mediate human AD is uncertain.

Some bacteria produce extracellular amyloid fibers that can stimulate innate immunity or influence protein aggregation in experimental systems. Direct cross-seeding of human Aβ or tau by microbial amyloid in sporadic AD remains a hypothesis, not an established human mechanism. Similarly, detection of microbial products in postmortem tissue is vulnerable to contamination and does not by itself prove a brain microbiome.

### 8.3. Evidence from Human Studies and Its Limitations

Several case–control studies report altered diversity or relative abundance of bacterial taxa in AD [109,112,113]. Associations have also been described among pro-inflammatory taxa, peripheral cytokines, endothelial dysfunction, circulating SCFAs or lipopolysaccharide, amyloid imaging, and cognition [109,110]. Yet taxonomic findings are inconsistent. Collection time, storage, DNA-extraction method, reagent contamination, sequencing platform or 16S rRNA gene region, batch structure, taxonomic and strain-level resolution, compositional-data analysis, geography, diet, medications, stool transit time, diagnostic criteria, disease stage, and sample size can each alter results [114,115]. Negative controls, randomized sample processing, batch-aware analyses, absolute-abundance or functional measurements when feasible, protocol harmonization, and complete reporting under microbiome-specific standards are necessary for reproducibility [115]. There is no validated AD microbiome signature suitable for diagnosis or treatment selection.

Transfer experiments strengthen biological plausibility but do not close the causal argument in humans. Microbiota from patients with AD impaired hippocampal-neurogenesis-dependent behavior when transplanted into microbiota-depleted young rats, and effects related to donor clinical measures [116]. Other mouse studies show that germ-free conditions, antibiotics, or microbiota transfer can modify amyloid pathology and microglial responses [117]. These experiments demonstrate that microbial communities can influence phenotypes in model systems. They do not prove that dysbiosis initiates AD in humans, because recipient physiology, model genotype, antibiotic exposure, and cross-species engraftment create artificial conditions.

Metabolomic and genetically informed analyses can identify candidate mediators, but their interpretation remains sensitive to confounding, reverse causation, multiple testing, and underlying model assumptions [110,111,114]. Longitudinal human cohorts with repeated stool sampling, detailed diet and medication measurement, AD biomarkers, and incident cognitive outcomes remain uncommon. The central unresolved question is not whether gut-derived signals can influence the brain, but whether a reproducible microbial function contributes sufficiently to human AD progression to constitute a useful, safe, and targetable mediator. Causal inference requires convergent designs. Priorities include prospective cohorts with repeated microbiome and AD-biomarker measurements before clinical progression, within-person analyses that reduce stable confounding, mechanistically specified interventions with verified ecological or metabolic target engagement, and replication across laboratories and populations. Germ-free or transplantation models can test biological sufficiency in a model system, but they cannot establish necessity or treatment efficacy in humans.

### 8.4. Microbiome-Directed Interventions

Dietary patterns rich in diverse plant fiber can alter microbial metabolism and improve cardiometabolic health, but any cognitive benefit cannot be attributed solely to the microbiome. Probiotics and synbiotics have produced preliminary cognitive or metabolic findings in small trials, including a 12-week study in AD [118]. Systematic reviews identify substantial heterogeneity in strains, dosing, duration, disease stage, cognitive outcomes, and risk of bias [114,119]. These products are not interchangeable, and current evidence does not support routine probiotic treatment as a disease-modifying therapy for AD.

Prebiotics, postbiotics, targeted metabolites, bacteriophages, and engineered microbes may offer greater mechanistic precision than nonspecific probiotic mixtures, but they remain early in development. Fecal microbiota transplantation (FMT) has a defined clinical role for recurrent Clostridioides difficile infection, not AD. Human AD evidence is limited to anecdotal or very small uncontrolled experience, while donor-dependent effects, pathogen transmission, antimicrobial-resistance genes, and long-term ecological consequences create meaningful risks [114]. FMT should not be used for AD outside rigorously regulated trials. A decisive microbiome trial would require biomarker-confirmed disease, a standardized intervention, verified ecological or metabolic target engagement, careful control of diet and medication, adequate duration, and prespecified cognitive and biological outcomes. Stratification may be essential because baseline microbiome and host genotype can modify response. Without these features, a negative trial is difficult to interpret and a positive cognitive signal remains vulnerable to bias. The gut–brain axis is a biologically plausible modifier pathway that has not yet yielded a validated AD biomarker or treatment.

## 9. Conclusions and Perspectives

AD is temporally ordered but biologically heterogeneous. Human genetic evidence provides the strongest support for an upstream role of Aβ. Pathogenic variants in *APP*, *PSEN1*, and *PSEN2* cause autosomal dominant AD. By contrast, *MAPT* variants cause primary tauopathies rather than AD. Regional tau burden is more closely associated with neurodegeneration and clinical progression than amyloid burden. Microglial and astrocytic responses, synaptic dysfunction, cerebrovascular impairment, bioenergetic deficits, defective proteostasis, and coexisting TDP-43, α-synuclein, and vascular pathologies further influence disease progression and clinical heterogeneity. Age-related decline in cellular maintenance and stress responses may increase vulnerability to these interacting mechanisms, linking AD to geroscience without defining it as an inevitable consequence of aging. Plasma p-tau217 and validated plasma Aβ42/40 assays can support biological assessment in symptomatic patients, although their interpretation depends on assay performance, clinical context, pretest probability, and comorbidities that affect biomarker concentrations. Anti-Aβ monoclonal antibodies modestly slow clinical decline in early symptomatic AD, but ARIA risk, which is higher in *APOE* ε4 carriers, and substantial implementation demands constrain their clinical impact. Gut microbiome alterations remain candidate systemic modifiers without established therapeutic relevance in humans. Further progress will require biomarker-guided patient selection and stage-matched therapies directed at mechanisms that remain active in the treated population.

## Figures and Tables

**Table 1:** The eight hallmarks of neurodegenerative diseases in Alzheimer’s disease.

Hallmark	References	Main Manifestations	Measures	Translational Interpretation
Pathological protein aggregation	[11,52,53,54,55,56,78,79,80,81,82,83,102,103]	Aβ plaques, cerebral amyloid angiopathy, tau seeds and tangles	Amyloid PET, CSF/plasma Aβ42/40, CSF/plasma p-tau, tau PET	Disease-defining biology, assembly state and location matter
Synaptic and network dysfunction	[11,12,13,14]	Synapse loss, hyperexcitability, disconnection	Cognitive and digital measures, EEG/MEG, FDG-PET, CSF synaptic proteins	Closely related to function but not specific to AD
Aberrant proteostasis	[72,73]	Endosomal, autophagic, lysosomal, and proteasomal dysfunction	Trafficking and lysosomal markers	Strong mechanistic rationale, limited validated clinical markers
Cytoskeletal abnormalities	[9,10,11,52,53,54,55,56,59,72,73,74,75,81,82,83]	Tau-mediated microtubule dysfunction, dystrophic neurites, impaired axonal transport	Tau biomarkers, NfL	Tau is central, but cytoskeletal injury extends beyond tangles
Altered energy homeostasis	[16,48,49,66,67,68,69,70,71,72,73,74,75,99]	Mitochondrial dysfunction, oxidative stress, insulin-signaling deficits, and vascular- metabolic dysfunction	FDG-PET, metabolic and vascular measures	Sometimes modifiable, causal specificity varies
DNA and RNA defects	[11,17]	DNA damage/repair deficits, epigenetic drift, RNA-processing changes	Multi-omics	Emerging, causal and clinical validation incomplete
Inflammation	[15,31,32,33,34,35,57,58,59,60,61,62,63]	Stage-dependent microglial and astrocytic states, complement and inflammasome signaling	GFAP, soluble TREM2, inflammatory PET	Response may be protective or harmful depending on context
Neuronal cell death	[10,11,18,52,53,54,55,56,81,82,83,102,103]	Neuronal and axonal loss, brain atrophy	MRI, NfL, total tau, neurodegeneration imaging	Late and nonspecific, indicates advanced injury

## Data Availability

No new data were created or analyzed in this study. Data sharing is not applicable to this article.

## Abbreviations

The following abbreviations are used in this manuscript:

Aβ	amyloid-β
AD	Alzheimer’s disease
ADP	adenosine diphosphate
APOE	apolipoprotein E
APP	amyloid precursor protein
ARIA	amyloid-related imaging abnormalities
AT(N)	amyloid, tau, and neurodegeneration
ATP	adenosine triphosphate
BACE	β-site amyloid precursor protein-cleaving enzyme
BBB	blood–brain barrier
CDR-SB	clinical dementia rating-sum of boxes
CSF	cerebrospinal fluid
Cyt c	cytochrome c
DNA	deoxyribonucleic acid
EEG	electroencephalography
FDG-PET	fluorodeoxyglucose positron emission tomography
FINGER	Finnish Geriatric Intervention Study to Prevent Cognitive Impairment and Disability
FMT	fecal microbiota transplantation
GFAP	glial fibrillary acidic protein
GWAS	genome-wide association study
LATE	limbic-predominant age-related TDP-43 encephalopathy
LDL	low-density lipoprotein
*MAPT*	microtubule-associated protein tau
MEG	magnetoencephalography
MRI	magnetic resonance imaging
NAFLD	non-alcoholic fatty liver disease
NfL	neurofilament light
NIA–AA	National Institute on Aging–Alzheimer’s Association
PET	positron emission tomography
PSEN	presenilin
p-tau	phosphorylated tau
RNA	ribonucleic acid
ROS	reactive oxygen species
SASP	senescence-associated secretory phenotype
SCFAs	short-chain fatty acids
TCA	tricarboxylic acid
TDP-43	transactive response DNA-binding protein 43 kDa
TREM2	triggering receptor expressed on myeloid cells 2
Ub	ubiquitin
